# Common variable immunodeficiency—an independent risk factor for atherosclerotic cardiovascular diseases

**DOI:** 10.3389/fcvm.2023.1289675

**Published:** 2023-11-08

**Authors:** Juho Mattila, Niina Pitkänen, Hannu Järveläinen

**Affiliations:** ^1^ Institute of Biomedicine, University of Turku, Turku, Finland; ^2^ Auria Biobank, Turku University Hospital and University of Turku, Turku, Finland; ^3^ Department of Internal Medicine, Satasairaala Central Hospital, Satakunta Hospital District, Pori, Finland

**Keywords:** CVID, common variable immunodeficiency, hypogammaglobulimenia, atheroschlerosis, atherosclerosis risk factor

## Abstract

Atherosclerosis, a disease of chronic inflammation of the arterial wall, is the main cause of most cardiovascular diseases (CVDs). Common variable immunodeficiency (CVID), a group of diseases characterized by frequent infections due to defective antibody production and lack of human immunoglobulins, plays a role in immune activation and inflammation. Thus, it can be hypothesized that CVID increases the risk for atherosclerotic CVDs. On the other hand, it is also possible that CVID patients are protected from atherosclerotic CVDs based on their life-long immunoglobulin therapy. Here, we examined whether patients with CVID have an increased risk for atherosclerotic CVDs or whether they are protected from these diseases. Using an electronic patient database registry search of a population of 83 CVID patients and their age- and sex-matched, tenfold larger control population we demonstrate that CVID patients have a statistically significantly higher risk for coronary heart disease (OR 2.4, *p* = 0.015) and peripheral vascular disease (OR 12.5, *p* < 0.001). Regarding cerebrovascular disease, there was a trend towards CVID patients having more strokes or ischemic attacks, but the difference was not statistically significant (OR 2.0, *p* = 0.133). The combined OR for CVID patients for atherosclerotic CVDs was 2.6 (*p* = 0.001). CVID population had more hypertension, but smoking was more seldom. There were no statistically significant differences in the incidence of diabetes or levels of serum total, HDL or LDL cholesterol, or glycosylated hemoglobin A1c between CVID patients and their controls. CVID patients had infections more frequently and the OR for autoimmune diseases was 3.8 (*p* < 0.001). Finally, a multivariate logistic analysis showed that CVID is an independent risk factor for atherosclerotic CVDs (*p* = 0.002). The present study demonstrates for the first time that CVID is an independent risk factor for atherosclerotic CVDs. Further studies are required to fully understand the exact mechanisms behind this.

## Introduction

Common variable immunodeficiency (CVID) is a heterogenous group of diseases characterized by frequent infections due to defective antibody production and lack or low levels of immunoglobulins. CVID is associated with high rates of co-morbidities such as lymphomas and other malignancies, chronic lung disease and autoimmune diseases like autoimmune hepatitis and inflammatory bowel disease (1–3). The standard treatment of CVID is regular intravenous immunoglobulin therapy given throughout life (4, 5). This aims at preventing acute and serious bacterial infections.

Atherosclerosis, the underlying cause of most cardiovascular diseases (CVDs), is defined as a disease of chronic inflammation (6, 7). The well-known risk factors for atherosclerosis include hypercholesterolemia, hypertension, hyperglycemia, genetic factors and smoking. Interestingly, a small study by Viera et al. (8) suggests that CVID should also be considered a risk factor of atherosclerosis via inflammation and dyslipidemia. Indeed, CVID patients have higher levels of inflammatory markers such as C-reactive protein (CRP) and tumor necrosis factor-alpha (TNF-α) and their high-density lipoprotein cholesterol and apoprotein A-I levels are lower than in controls (8). The lipid profile of CVID patients has been proven to be disturbed with low levels and function of high-density lipoprotein (HDL), high levels of triglycerides and very low-density lipoprotein (VLDL) and an unfavorable fatty acid profile (9).

Furthermore, multiple studies have suggested that infections should also be considered as an important risk factor for atherosclerotic CVDs (10). By definition, patients with CVID have more frequent infections. It has also been shown that patients with acquired immunodeficiency (HIV) tend to have a significantly higher risk for developing atherosclerosis (11).

CVID-patients are well known to be at high risk of developing autoimmune diseases and other inflammatory conditions (1, 12), which cause abnormal T-cell, monocyte and macrophage function (13–15). These have been linked to low grade systemic inflammation, which is known to contribute to the development of atherosclerosis (16–19).

However, the relationship between CVID and atherosclerosis remains to be resolved (20). Experimental animal studies have shown that frequent treatment with immunoglobulin infusions drives a protection against the development of atherosclerosis (21). Reduced IgG-levels have also been shown to cause endothelial dysfunction, which can be improved by immunoglobulin infusions (22). Thus, it can be hypothesized that frequent immunoglobulin therapy may protect CVID patients from developing atherosclerosis.

Here, we aimed to examine whether patients with CVID have an increased incidence of atherosclerotic CVDs or whether they are protected from these diseases. The study was performed by conducting an electronic patient database registry search of a population of 83 CVID patients and their age- and sex matched, tenfold larger control population.

## Materials and methods

Patients with diagnosed CVID were identified by searching for ICD-10 (International Classification of Diseases) code D80 from the electronic patient database of Turku University Hospital in Southwestern Finland. The total number of patients with CVID diagnosis in our study was originally 114. The diagnosis of each patient was confirmed by reading through their medical history. The diagnosis of the patients was based on two major criteria, i.e., increased number of infections before the diagnosis or severe septic infections and low serum immunoglobulin levels (IgA, IgG, IgM and their subtypes). The total number of patients accepted in this study was 83. Patients with secondary or transient immunodeficiency or false diagnosis were ruled out. Patients who had moved out of Turku University Hospital area were also excluded due to restricted access to their later medical history.

For each patient diagnosed with CVID, ten age and gender-matched control patients (*N* = 830) were randomly selected from Turku University Hospitals' patient registry. None of the control patients were diagnosed with CVID. The patient registry includes the history and diagnoses of all the patients who have visited public hospitals in Southwestern Finland during 2004–2017. The diagnosis, treatment and follow-up of CVID patients were carried out in public hospitals in Southwestern Finland, primarily in the units of infectious diseases. All public hospitals in Southwestern Finland use the same Turku University Hospital patient database.

The lowest immunoglobulin levels measured for each patient were noted. Also, the duration of disease and the duration of immunoglobulin treatment were noted. Laboratory records of the known major risk factors for atherosclerosis were extracted from the patient database. These risk factors included serum lipid levels and glycosylated hemoglobin A1c. Individual mean laboratory values were calculated for each patient and a group mean was calculated for CVID group and the control group, respectively. The smoking status of individuals in both the CVID group and the control group was extracted from the patient database with a search tool using a deep learning-based model (23). Smoking status could be extracted for 469 (=51.4%) of the patients (412 controls, 57 CVID patients). To rule out the role of hypertension and diabetes in the development of atherosclerosis, the incidence of these two diseases was calculated for both groups.

A search for all ICD-10 codes was conducted for all the patients in the CVID and the control populations. ICD-10 codes used for atherosclerotic cardiovascular diseases included I20 (angina pectoris), I21 (ST-elevation or non-ST-elevation myocardial infarction), I25 (chronic ischemic heart disease), I63 (cerebral infarction), I67 (other cerebrovascular disease), I70 (peripheral vascular disease). The number of patients detected with the above diagnoses were calculated in both CVID and control populations. The risk for these atherosclerotic CVDs was calculated for both groups as well as a combined risk.

As CVID patients are immunocompromised and thought to have infections more frequently, data on the patients' ICD-10 codes for infections were retrieved and compared to those of the control population. Many autoimmune conditions also cause inflammation, so the data on the prevalence of inflammatory autoimmune diseases were also gathered for both the CVID and control groups using the ICD-10- codes.

## Statistical methods

Comparison of continuous variables between groups was done using Student's *t* test or Mann-Whitney test. Fisher's exact test was used to analyze differences between categorical variables. Association of CVID with the risk for CVD was analyzed using logistic regression. Multivariable logistic regression model including CVID, age, sex, hypertension and smoking status was used to assess the effect of CVID on CVD risk independently of other risk factors. All statistical analyses were performed using Python version 3.6.10.

## Results

The number of patients included in the study was 83 (72.8%) out of 114 patients with CVID diagnosis (Table 1). Excluded patients included nine (7.9%) typing errors, six (5.3%) patients who had moved to other districts, six (5.3%) patients whose immunodeficiency was caused by the treatment of lymphomas, four (3.5%) patients with treatment of chronic lymphocytic leukemia and one (0.9%) patient with the treatment of acute myelocytic leukemia. Two (1.8%) patients only had suspected immunodeficiency, but the diagnosis was never confirmed. One (0.9%) patient had a transient immunodeficiency caused by severe malnutrition. One (0.9%) patient had immunodeficiency that was considered to be a side-effect of phenytoin. One (0.9%) patient visited Turku University Hospital only for a single immunoglobulin infusion. Of these patients 13 (11.4%) had secondary immunodeficiency.

**Table 1:** Demographics of the CVID-patients.

CVID patients	
Patients included (*N*/%)	83 (72.8%)
Mean age (year)	53.7
Male (*N*)	32
Female (*N*)	51
Alive (*N*/%)	66 (79.5%)
Deceased (*N*/%)	17 (20.5%)
Mean duration of disease (year)	14.8
Immunoglobulin therapy (*N*/%)	77 (92.8%)
Mean duration of immmunoglobulin therapy (year)	13.1
p-IgA mean (g/L)	0.71
p-IgM mean (g/L)	1.15
p-IgG mean (g/L)	4.14
p-IgG4 mean (g/L)	0.13
Vaccine response tested (*N*/%)	38 (45.8%)
Inadequate response for vaccines (*N*/%)	31 (81.6%)

**Figure 1:**
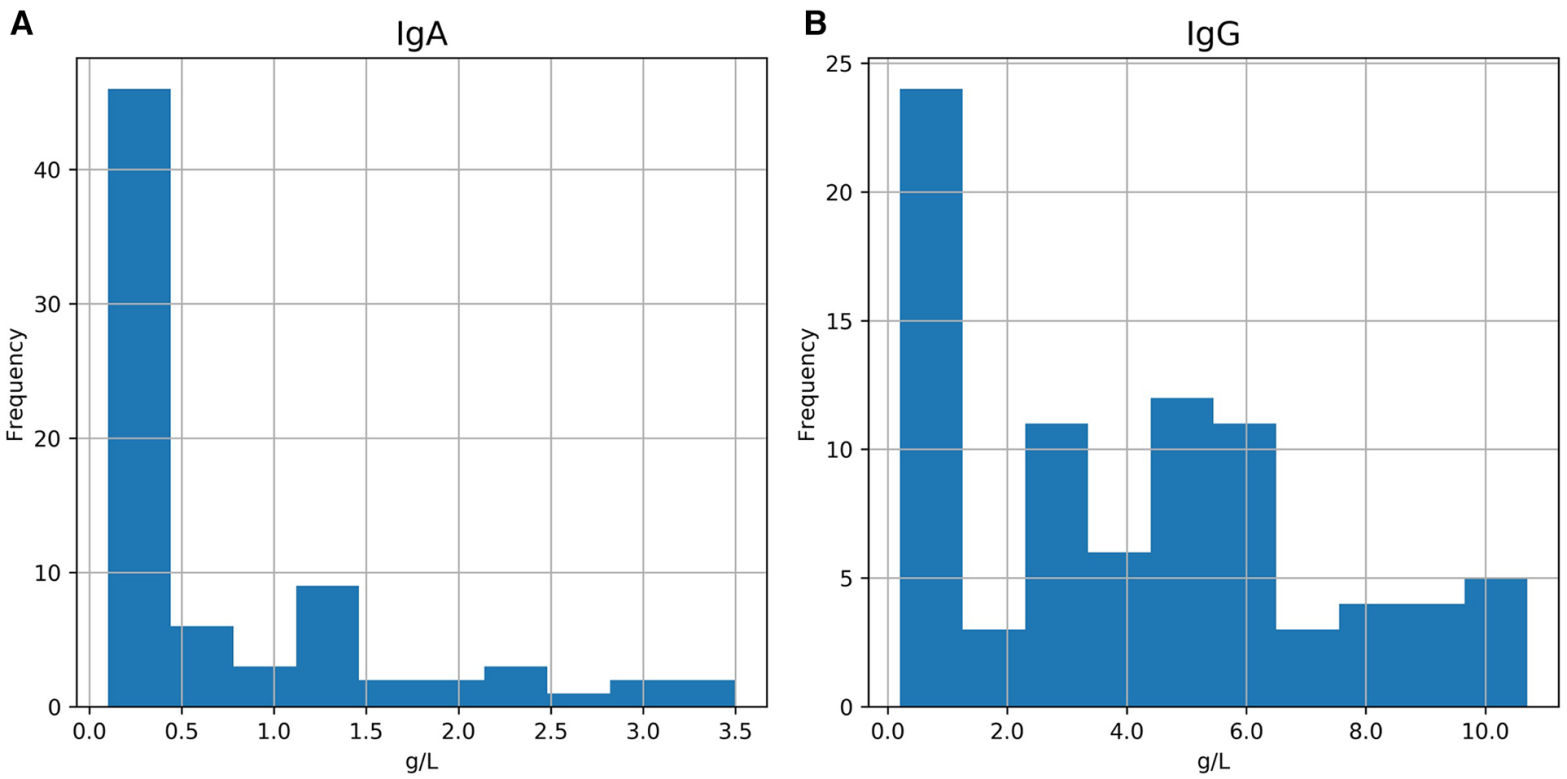
The IgA (**A**) and IgG (**B**) distribution of the patients in the CVID group. IgA min. was 0.1 g/L and max. was 3.5 g/L. IgG min. was 0.2 g/L and max. was 10.7.

The age and gender of the patients with CVID at the date of data extraction (17.4.2017) are shown in Table 1. Of the 83 patients included 66 (79.5%) were alive and 17 (20.5%) were deceased. The mean duration of their illness was 14.8 years. The mean IgA levels of our CVID population was 0.71 g/L, the mean IgM levels 1.15 g/L, and the mean IgG-levels 4.1 g/L. IgA and IgG distributions for CVID group are shown in Figure 1. Of the included patients, 77 (92.8%) had received immunoglobulin-infusions. The mean duration of immunoglobulin treatment was 13.1 years (Table 1).

For 38 (45.8%) patients in CVID group, vaccine response was assessed with vaccines against Streptococcus pneumoniae, Corynebacterium diphtheriae, Clostridium tetani and Bordetella pertussis. Of the tested patients, 31 (81.6%) had inadequate vaccine response (Table 1).

Patients in the CVID group had significantly higher incidence of coronary heart disease compared to subjects in the control population (13.25% vs. 6.05%, OR 2.4, *p* = 0.015). The incidence of peripheral vascular disease was also significantly higher in the CVID group than in the control group (9.64% vs. 0.85%, OR 12.5, *p* < 0.001). Regarding cerebrovascular disease, there was a similar trend towards CVID patients having more strokes or ischemic attacks, but the difference was not statistically significant (OR 2.0, *p* = 0.133). The combined incidence of any event of the above was also calculated to evaluate the total incidence of atherosclerotic CVDs; CVID group had a 21.69% incidence of CVDs while the incidence in the control group was 9.55%. As such, the risk for atherosclerotic CVDs was significantly higher in the CVID group than in the control group (OR 2.6, *p* = 0.001) (Table 2).

**Table 2:** Combined risk for atherosclerotic disease for CVID patients compared to control group.

Disease	ICD-codes	CVID *N* (%)	Control *N* (%)	OR (95% CI)	*p*
Coronary heart disease	I20–I25	11 (13.25)	50 (6.05)	2.4 (1.2–4.8)	0.015
Peripheral atherosclerosis	I70, I71, I74	8 (9.64)	7 (0.85)	12.5 (4.4–35.4)	<0.001
Cerebrovacular disease	I63–I65, I67, G45, G46	6 (7.23)	31 (3.75)	2.0 (0.8–4.9)	0.133
Any of the above	Any of the above	18 (21.69)	79 (9.55)	2.6 (1.5–4.6)	0.001

The most common risk factors for atherosclerotic CVDs for the CVID group and the control group are shown in Table 3. Hypertension was diagnosed in 22.9% of the patients in the CVID group and in 13.4% of the patients in the control group (*p* = 0.031). The number of patients diagnosed with diabetes was higher in CVID patients (10.8%) than in controls (7.4%), but the difference was not statistically significant. Smoking was more common in the control group (35.0%) than in the CVID group (19.3%), (*p* = 0.023). The median CRP calculated for each individual was slightly higher in the control group than in the CVID group (6.0 vs. 10.5, *p* = 0.017). There was no statistically significant difference in the serum lipid levels or median blood glycosylated hemoglobin A1c levels. Finally, in a logistic regression model adjusted for age, sex, smoking status, hypertension, history of autoimmune disease and median CRP, CVID remained an independent risk factor for atherosclerotic CVDs (OR: 2.00, 95% CI: 0.90–4.40, *p* = 0.002) (Table 4). A subgroup analysis was done for risk of any atherosclerotic disease between CVID patients who received immunoglobulin infusions and CVID patients who did not receive immunoglobulin infusions, but there was no statistically significant difference in the risk (*p* = 0.479).

**Table 3:** Risk factors for atherosclerotic disease for CVID group and control group. Values are mean ± SD for normally distributed variables and median (25th–75th percentile) for non-normally distributed variables.

Risk factor	CVID		Control		*p*
		N		N	
Male gender (*N*/%)	32 (38.6)	83	319 (38.5)	828	1
Age (year)	53.7 ± 29.5	83	53.6 ± (20.2)	828	0.986^a^
Hypertension (*N*/%)	19 (22.9)	83	111 (13.4)	827	0.031^b^
Diabetes (*N*/%)	9 (10.8)	83	61 (7.4)	827	0.277^b^
Any autoimmune disease (*N*/%)	40 (48.19)	83	119 (14.39)	827	<0.001^b^
Smoking (*N*/%)	11 (19.3%)	57	144 (35.0%)	412	0.023^b^
GHbA1c%	5.60 (5.31–6.21)	30	5.78 (5.5–6.6)	103	0.061^c^
Cholesterol (mmol/L)	4.61 ± 0.98	43	4.91 ± 1.03	167	0.092^a^
HDL (mmol/L)	1.39 ± 0.55	41	1.49 ± 0.50	164	0.228^a^
LDL (mmol/L)	2.50 ± 0.72	41	2.69 ± 0.90	153	0.21^a^
Triglycerides (mmol/L)	1.35 (1.0–1.8)	44	1.35 (0.95–2.05)	163	0.45^c^
CRP (mg/L)	6.0 (3.0–18.0)	81	10.5 (3.0–41.0)	428	0.017^c^

^a^ *t*-test.

^b^ Fisher's exact test.

^c^ Mann-Whitney test.

**Table 4:** A logistic regression model adjusted for age, sex, smoking status, hypertension, history of any autommune disease and median CRP. CVID remained an independent risk factor for atherosclerosic CVDs.

Risk factor	OR (95% CI) *N* = 469	*p*
CVID	2.00 (0.90–4.47)	0.002
Age	1.08 (1.05–1.11)	<0.001
Sex	2.82 (1.49–5.37)	0.002
Smoking	1.22 (0.61–2.42)	0.57
Hypertension	2.44 (1.31–4.57)	0.005
Autoimmune diseases	1.31 (0.67–2.54)	0.428
Median CRP	1.0 (0.99–1.01)	0.803

There were statistically significant differences in the rates of infections between the two groups (Table 5). CVID patients had more gastrointestinal infections (OR 3.8, *p* < 0.001), viral diseases (OR 8.6, *p* < 0.001), dermal infections (OR 4.8, *p* < 0.001), respiratory infections (OR 10.9, *p* < 0.001, urogenital infections (OR 3.9, *p* < 0.001), septicaemias (OR 14.2, *p* < 0.001, eye infections (OR 3.5, *p* < 0.019), and otolaryngologistic infections (OR 4.5, *p* < 0.001).

**Table 5:** Rates of infections for CVID group and control group.

Type of infection	ICD-10-codes	CVID *N* (%)	Control *N* (%)	OR (95% CI)	*p*
GI-infections	A04-A09, K12-K81	48 (57.8)	219 (26.5)	3.8 (2.4–6.0)	<0.001
Viral diseases	A60, B00-B27	10 (12)	13 (1.6)	8.6 (3.6–20.2)	<0.001
Dermal infections	A46, B35-B37, L02, L03, L08	16 (19.3)	39 (4.7)	4.8 (0.6–9.1)	<0.001
Respiratory infections	J01-J40	63 (75.9)	186 (22.5)	10.9 (6.4–18.4)	<0.001
Urogenital infections	N10-N30	15 (18.1)	44 (5.3)	3.9 (2.1–7.4)	<0.001
Sepsis	A41	9 (10.8)	7 (0.8)	14.2 (5.2–39.4)	<0.001
Eye infections	H10, H16	5 (6)	15 (1.8)	3.5 (1.2–9.8)	0.019
Otolaryngologistic infections	H60, H66	9 (10.8)	22 (2.7)	4.5 (2.0–10.0)	<0.001
CNS-infections	G00	2 (2.4)	0		
Osteomyelitis	M86	1 (1.2)	1 (0.1)	10.1 (0.6–162.5)	0.104

Patients in the CVID group had statistically significantly more autoimmune diseases than patients in the control group; the OR for any inflammatory autoimmune disease was 5.5 (*p* < 0.001). CVID patients had higher incidence of sarcoidosis (OR 3.8, *p* = 0.05), autoimmune thyroid diseases (OR 5.1 *p* < 0.001), asthma (OR 7.6, *p* < 0.01), inflammatory bowel diseases (OR 8.4, *p* < 0.001), autoimmune mediated liver or biliary disease (OR 10.4, *p* = 0.001) and rheumatoid disease (OR 2.9, *p* = 0.003). In contrast, there was no statistically significant difference in the incidence of type I diabetes.

In our data the prevalence of CVID in Southwestern Finland was 14/100,000 total; 11.3/100,000 for males and 16.5/100,000 for females.

## Discussion

To our knowledge, this is the first study directly comparing the risk for CVDs between CVID patients and patients not suffering from CVID. We have shown here that patients with CVID are more likely to be diagnosed with atherosclerotic CVDs, i.e., coronary heart disease, peripheral vascular disease and cerebrovascular disease. The logistic multivariate analysis showed that CVID is an independent risk factor for atherosclerotic CVDs.

It has earlier been shown that patients with rheumatoid disease or inflammatory bowel disease have increased CVD mortality (24–27); the hypothesis behind this is the ongoing autoimmune inflammation. We also found out that CVID patients in this study had more frequent inflammatory autoimmune conditions, which is in line with previous studies (28–31) The multivariable logistics model showed that the increased risk for atherosclerosis is not only due to increased autoimmune burden. These findings emphasize the key role of inflammation in the development of atherosclerosis (6).

The prevalence of CVID in this study was 14/100,000 which is surprisingly high compared to previous studies in Finland (32). Another recent study conducted in the South and Southeastern Finland showed a prevalence of 5.5/100,000 (28). There is a great variance in the prevalence of CVID in different countries ranging from 3.8/100,000 in Denmark to 0.1/100,000 in Spain (28). The heterogeneity of the study population may explain this unusually high prevalence.

The limitations of the present study include its retrospective design. The diagnoses were retrieved from the electronic patient database of Turku University Hospital, but there is always the chance of misdiagnoses, or missing ICD-10 diagnoses. To reduce this risk the diagnoses of CVID were also manually checked and patients with secondary immunodeficiencies were ruled out. In Finland, we do not have a reliable national patient registry yet, so the patients who have moved out from the Southwestern Finland were also ruled out to avoid missing diagnoses.

Not all the patients in the CVID population of this study meet the current ESID (European Society of Immunodeficiencies) diagnostic criteria for CVID (33) as the vaccine response had not been tested for the entire population. Some of the patients in the study population had only decreased IgG-levels and so would be classified as “possible CVID” even on ESID 1999 -criteria (34). Data on lymphosyte immunophenotyping was limited for the CVID-group. However, the CVID diagnoses of the present study can be regarded as correct, because all of the patients have been followed in the same unit, namely Turku University Hospital's outpatient clinic by specialists in infectious diseases rather than in primary healthcare. The immunoglobulin range shown in Figure 1. reveals that 16 patients (19.3%) in the CVID-population have had IgG-levels higher than 6.8 g/L in our data. Most of these patients had CVID diagnosed earlier than 2004, and data on the IgG-levels at the time of diagnosis is missing. IgG-levels measured for these patients was during immunoglobulin replacement therapy. Some of these patients were diagnosed with IgG-subclass deficiency only but had received immunoglobulin infusions due to increased number of infections and inadequate vaccine response.

We did not differentiate between subgroups of CVID like more rare monogenetic defects or possibly milder antibody defects due to small sample size. It remains unclear whether patients with monogenic antibody patients have even higher risk for atherosclerosis. This could be an interesting topic for future research; this would eliminate the heterogeneity of our cohort that may impact the results presented in this study.

Nearly all of the patients have been treated with immunoglobulins for the deficiency. Some patients have declined from immunoglobulin treatment, but the included patients still have had scheduled visits in the outpatient clinic for follow-up. Due to small sample size, there was no statistically significant difference in the atherosclerosis risk between CVID patients treated with immunoglobulins and those CVID patients who did not receive immunoglobulin replacement therapy. Our study does not clarify the role of immunoglobulin infusions in preventing the development of atherosclerosis. Thus this needs further research. It is also possible that atherosclerotic vascular disease is more likely to be diagnosed as CVID patients are more exposed to clinical care than control patients, which could bias the results.

The strengths of this study include quite a large population and a large age- and sex-matched control group. The confounding factors such as other traditional CVD risk factors have been ruled out. In this study, there were no statistically significant differences in the levels of serum total cholesterol, HDL or LDL cholesterol, which is different from the results by Vieira et al. (8). This may be due to the large number of controls in this study.

We have shown that patients with CVID have statistically significantly increased risk for developing atherosclerotic CVDs. The hypothesis is that this is due to increased number of infections and ongoing inflammation. This study indicates that clinicians treating patients with CVID should pay more attention to the primary prevention of atherosclerotic CVDs and to actively seek for symptoms of CVDs as the risk of these patients for CVDs is significantly higher than that of the control population.

In summary, in this study we have shown that CVID is an independent risk factor for atherosclerotic CVDs. Further studies are required to find out the exact mechanisms of atherosclerotic CVDs in patients with immunodeficiencies as well as the importance of immunoglobulin supplementation in CVD risk.

## Data Availability

The raw data supporting the conclusions of this article will be made available by the authors, without undue reservation.
